# Preliminary Study on the Clinical Significance and Methods of Using Carbon Nanoparticles in Endoscopic Papillary Thyroid Cancer Surgery

**DOI:** 10.1155/2021/6652315

**Published:** 2021-04-26

**Authors:** Shangrui Rao, Zhonglin Wang, Congtao Pan, Yi Wang, Zhe Lin, Zhongliang Pan, Jian Yu

**Affiliations:** Department of General Surgery, Wenzhou Central Hospital, Wenzhou 325000, Zhejiang, China

## Abstract

**Purpose:**

The purpose of this study was to find the clinical significance and methods of using CN in endoscopic treatment for PTC.

**Materials and Methods:**

A total of 108 cases were randomly enrolled and divided into two groups, with 50 cases in the CN injection group who were injected with CN and 58 cases in the control group with no CN injection. All cases were analyzed with the size of carcinoma, the number of lymph node, and parathyroid gland injury.

**Results:**

All operations were successfully completed. The lymph node dissection number was 274 for the control group and 322 (the rate of black stained was 87%) for the CN injection group. The average number of lymph nodes in the CN injection group was 6.44 ± 2.08, which was significantly higher than that in the control group (4.72 ± 1.89). The control group had a relatively higher incidence of incidental parathyroidectomy, compared to the CN injection group (27.6% in the control group vs. 12% in the CN injection group, *P*=0.045). However, the incidence of hypoparathyroidism failed to show the significant difference between the two groups.

**Conclusion:**

Using CN in endoscopic PTC surgery could increase the detection rate of lymph nodes and reduce the injury of parathyroid glands to a certain extent.

## 1. Introduction

Thyroid cancer is considered the most prevalent endocrine cancer, especially in women [1–3]. Papillary thyroid cancer (PTC) accounts for 80–90% of all thyroid cancer [4–6]. The WHO defines papillary thyroid carcinoma as papillary thyroid microcarcinoma (PTMC), with a diameter of less than 1.0 cm, regardless of whether it has infiltration or lymph node metastasis or even distant metastasis [7]. As previously reported, 20–50% of PTC patients had cervical lymph nodes [8]. Routine prophylactic central neck dissection (CND) has been recommended by the Japanese Society of Thyroid Surgeons and the French Society of Otolaryngology Head and Neck Surgery [9]. However, expanding the scope of operation will inevitably bring corresponding complications, including hypoparathyroidism, recurrent laryngeal nerve (RLN) paralysis, RLN permanent damage, and even tracheal or esophageal damage.

The injection of a suspension of carbon nanoparticles (CN) comprises nano-sized carbon particles with an average diameter of 150 nm. CN have a characteristically high degree of lymphatic system tropism, fast tracing speed, and high rate of dyeing black with strong color contrast with the surrounding tissue [10]. CN have been applied in the protection of parathyroid glands (PGs) by staining lymph node into black except the PGs, which could help operators identify the PGs quickly.

Several studies confirm that CN facilitate lymph nodes dissection and PGs identification in conventional PTC surgery [11–15]. However, the application of CN in total endoscopic thyroidectomy (TET) for PTC is limited. As a new surgical technique, there are some disadvantages of TET, including two-dimensional operative views, the small and narrow working space, interference between surgical instruments, and lack of tactile sensation. Different from the traditional open surgery, CN injection in TET needs considerable techniques and skills. The present study reported the clinical significance and methods of using CN in TET.

## 2. Materials and Methods

### 2.1. General Information

A total of 108 PTMC patients who underwent TET from January 2015 to April 2019 in our hospital were selected. According to the random number table method, they were divided into the CN injection group (50 patients used CN during operation) and the control group (58 patients with no use of CN). The clinical characteristics of the patients enrolled in the study are given in Table 1. The study was filed and approved by the Ethics Committee of Wenzhou Central Hospital. Signed consent was obtained from all participants or their surrogates.

### 2.2. Inclusion and Exclusion Criteria

Inclusion criteria: (1) preoperative ultrasound puncture confirmed pathological diagnosis of thyroid cancer. (2) Lesions located in one glandular lobe and diameter less than 1.0 cm. (3) Preoperative cervical lymph node ultrasound that indicated cN0. (4) Patients had cosmetic requirements and agreed to perform TET surgery. (5) All operations were performed by surgeons in the same treatment team.

Exclusion criteria: (1) non-PTMC patients. (2) Bilateral glandular lesions. (3) Preoperative ultrasound and enhanced CT showed that cervical lymph nodes had metastasis. (4) No cosmetic requirements. (5) History of thyroid surgery. (6) History of cervical surgery and radiotherapy.

### 2.3. Surgical Procedure

Complete areola approach was used in all operative approaches. After the operation space was established by continuous subcutaneous CO_2_ filling, the white line of the neck was cut by an ultrasound knife to expose thyroid glands on the affected side.

**Figure 1:**
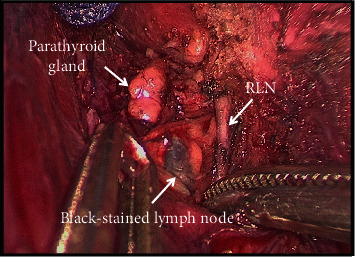
Black-stained lymph node, recurrent laryngeal nerve (RLN), and parathyroid gland under “negative imaging” seen during operation.

**Figure 2:**
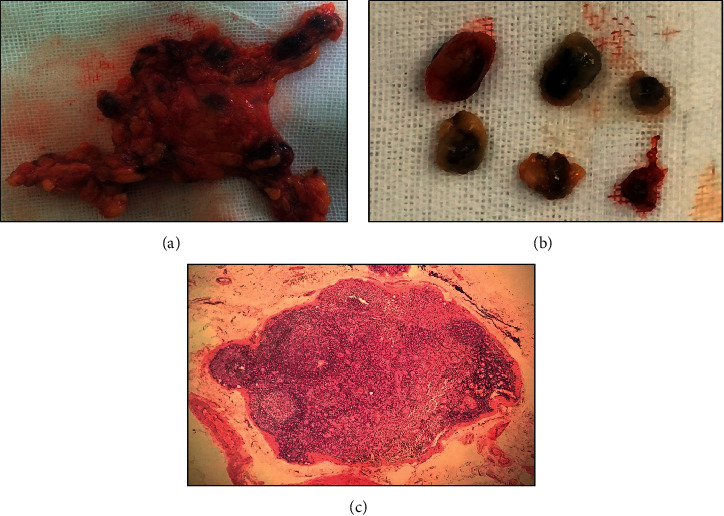
Specimens of lymph nodes with black staining and microscopic findings in the experimental group (H&E ×100).

In the CN injection group, the affected glands were stained with carbon after injection of CN (0.5 ml, Chongqing LUMMY Pharmaceutical Co., Ltd., suspended with sterile water), and then, the contralateral glandular lobes were exposed routinely. About 20 minutes later, the affected glandular lobes, isthmus, and central lymph nodes were routinely dissected. Black-stained lymph node, RLN, and PG under “negative imaging” were seen during operation (Figure 1). After operation, specimens were sent for routine pathological examination (Figure 2).

The control group was routinely treated with lobectomy, together with isthmus resection and central lymph node dissection.

### 2.4. Injection Method of CN

**Figure 3:**
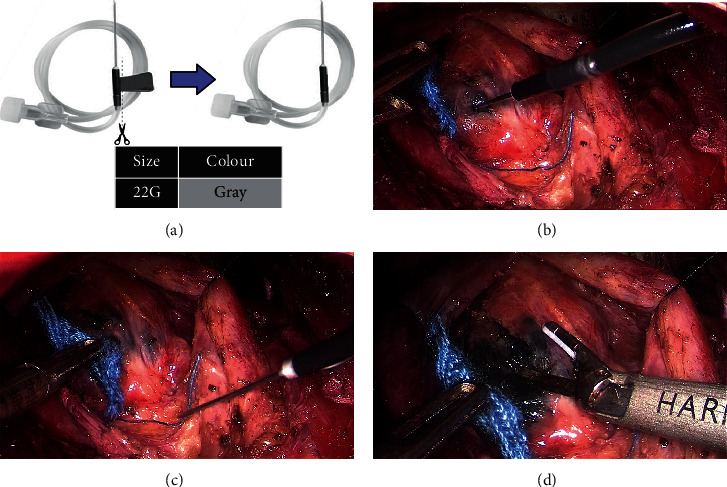
Method A: injected by trocar.

Injected by trocar (method A, Figure 3): (1) taking 22G scalp needle and cutting off its needle wings to make an improved scalp needle (Figure 3(a)). (2) Inserting the modified scalp needle into the operation area through the 5 mm trocar on the right side and injecting into the gland under direct vision (Figure 3(b)).

**Figure 4:**
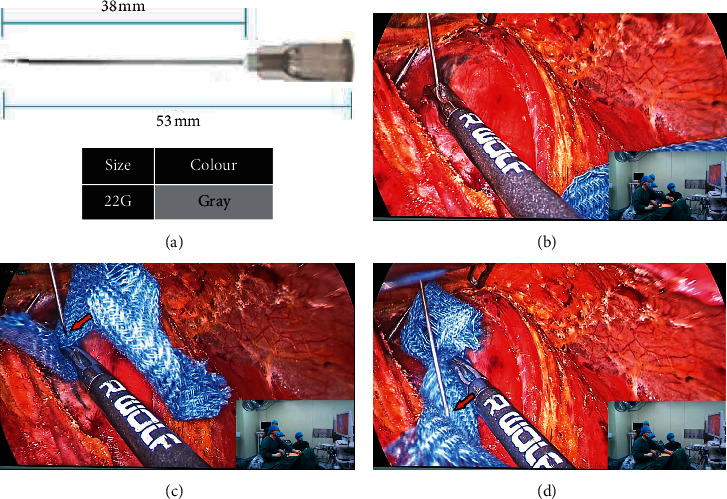
Method B: percutaneous puncture.

Percutaneous puncture (method B, Figure 4): (1) taking 1 ml syringe and replacing its needle with 22G needle (Figure 4(a)). (2) Direct percutaneous injection with 22G needle after gland exposure (Figure 4(b)).

**Figure 5:**
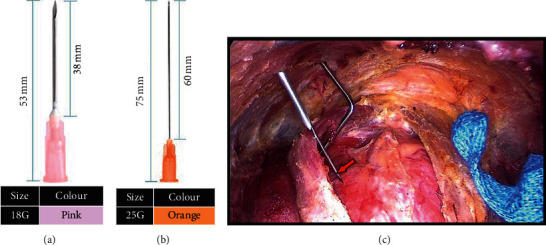
Method C: improved percutaneous puncture.

Improved percutaneous puncture (method C, Figure 5): (1) using the 18G needle (Figure 5(a)) to establish a tunnel at the anterior neck skin of the repuncture. (2) Selecting a thinner 25G needle (Figure 5(b)) and passing through the 18G needle (Figure 5(c)). (3) Injecting the CN into exposed glands.

### 2.5. Observation Index

The rate of RLN injury and parathyroid injury were recorded. All specimens were sent to the pathology department for examination. The total number of lymph node dissection and metastasis in the control group, the number of black-stained lymph nodes in the CN injection group, the number of nonblack-stained lymph nodes, and their respective metastases were counted, and the detection of PGs was analyzed.

### 2.6. Statistical Analysis

The *t*-test and chi-square test were used for the measurements and count data, respectively. *P* < 0.05 was considered to indicate statistical significance. For all statistical analyses, SPSS 19.0 package was used.

## 3. Results

Among all 108 patients, 50 cases were allocated into the CN injection group, and 58 cases were in the control group. The mean age for the CN injection group and the control group was 44 and 46, respectively. The baseline characteristics are given in Table 1. All operations were successfully completed, and none of them was transferred to open surgery.

A total of 274 lymph nodes were detected in the control group, with an average of 4.72 ± 1.89 in each case; 322 lymph nodes were detected in the CN injection group, with an average of 6.44 ± 2.08 in each case, of which 281 were black stained (the rate of black stained was 87%) (Table 1). Postoperative pathological specimens showed no significant difference in the tumor size between the two groups.

There was no significant difference between the two groups in the detection rate of PG and the rate of RLN injury (Table 2). Furthermore, no significant difference in thyroid and parathyroid functions between the two groups after operation was observed (Table 3).

## 4. Discussion

This study showed that the application of CN in endoscopic PTC surgery could increase the detection rate of lymph nodes and reduce the injury of parathyroid glands.

Endoscopy technology is the inevitable trend of modern surgery development. Since the endoscopic thyroidectomy (ET) was first reported in 1997 [16], various endoscopic approaches have been applied to thyroid surgery [17–19]. The applications were not only for benign thyroid tumors but also for the malignant ones [20]. PTMC constitute approximately 30% of all differentiated thyroid cancers and are largely responsible for the increased incidence of thyroid cancer in many countries over the past decade [21]. Previous research has indicated that endoscopic thyroid surgery is an effective alternative for selected patients with PTMC, compared with conventional open thyroid surgery [22, 23]. Wang et al. demonstrated that application of CN played a key role in protecting PGs and allows a thorough dissection of the central lymph nodes in endoscopic surgery. In addition, CN's application is an important factor for rapid recovery of parathyroid function [24].

Sywak et al. suggested that the routine ipsilateral level VI lymphadenectomy for PTC allowed a thorough clearance of thyroid tissue from the area at a high risk of metastasis [25]. Evidence indicated that prophylactic CND might prevent recurrence and improve overall survival [26]. Radionuclides and methylene blue have been applied for detection of the sentinel lymph node in patients with PTC. However, the use in the management of PTC is limited because of low sensitivity, insufficient stability, and a high false-negative rate [27–29]. As a novel type of lymphatic tracer, CN suspension has been widely used to assist cleaning the lymph nodes by dyeing them during breast, gastric, and thyroid surgery. In traditional open thyroidectomy, it has been proved that NC can improve the detection rate of lymph nodes, with a black staining rate ranging from 69.89% to 95.26% [11, 12]. The present study showed that this technique can also be performed safely in endoscopic surgery. In TET operation, combined with the magnification of high-definition endoscopy camera lens, the lymph nodes and surrounding tissues can be identified more clearly, especially for the detection of small and concealed lymph nodes. Sun et al. [12] and Zhu et al. [13] reported that more small lymph nodes, particularly those <2 mm, were detected, compared with the control group for whom CN were not used. The results of our study indicated that the endoscopic use of CN had a high black staining rate (89%), and the number of lymph nodes detected in the CN injection group was significantly higher than that in the control group. However, 3 obviously enlarged metastatic lymph nodes (diameter 1.5 ± 0.4 cm) were not stained by CN in the CN injection group, one of which was lymphatic vessel cancer thrombus. Considering that the lymphatic vessel was blocked and could not be stained by CN, this suggested that we should pay attention to the difference between “negative imaging” of PG. If necessary, quick pathological examination should be performed during the operation.

The incidence of hypocalcemia after operation due to temporary or permanent hypoparathyroidism secondary to iatrogenic parathyroid injury is as high as 5%–25%, which is often caused by inaccurate excision of the parathyroid gland or its blood supply disorder [30–32]. Exposure and confirmation of PGs can effectively reduce the incidence of hypoparathyroidism [33]. Previous studies have demonstrated that CN can be used to protect the parathyroid in conventional surgery [12, 14, 15]. However, it is difficult to identify PGs in TET due to the lack of tactile sensation and special vision, especially in patients with abnormal location of the inferior PG. Wang et al. [24] showed that PGs were present in the thyroid or central nodal specimens of five patients, which were all in the control group. And the control group had a relatively higher incidence of incidental parathyroidectomy compared to the CN injection group. The present study confirmed that CN could reduce the prevalence of PGs injury after TET (*P*=0.045).

The following aspects should be paid attention when using CN suspension. First, injection can be made after slightly exposing the thyroid glands on the affected side by incision of the white line of the neck to avoid excessive separation of glands affecting lymphatic drainage. Second, the method of injection by trocar (method A) is recommended to beginners. The gas in the pipe should be exhausted before injection. Third, it is easy to control the injection force by percutaneous puncture (method B). However, before entering and leaving the skin, it is necessary to clean the residual CN liquid on the injection needle (Figure 4(c)). The needle can be cleaned with small yarn or punctured on the surrounding muscle tissue for several times (Figure 4(d)). Otherwise, permanent pigmentation will be left on the neck skin. Fourth, the improved percutaneous puncture (method C), which can not only ensure the control of injection force but also avoid the pigment pollution of CN in the neck skin, can also be used. Fifth, the injection dose should not be too large. For unilateral PTMC surgery, we only need to use about 0.2 ml. Furthermore, the injection should not be too deep. Due to the lack of tactile sensation under endoscopy, beginners often control the needle instability, which can easily lead to injection too deep. In our study, we initially encountered a case of incorrect CN injection penetrating into the dorsal thyroid gland, which greatly interfered with the TET field of vision and could not clearly identify the RLN during the operation. Last, pullback after injection to prevent misinjection of blood vessels, keep the needle in a negative pressure state to absorb the exudate with small yarn in time, and use electric knife or ultrasonic knife head to close the puncture point on the gland (Figures 3(c) and 3(d)).

However, there are some limitations in this study. First, the sample size in this study is relatively small. Further evidence with a large sample size and extension of a wider range of participants is warranted to further verify the findings. Second, the size of postoperative lymph nodes is not measured, which may influence the detection rate of microlymph nodes in endoscopic thyroid surgery.

In summary, the results of this study indicate that the use of CN in TET for PTMC can increase the detection rate of lymph nodes and reduce the injury of PGs. However, the use of CN is different from open surgery, and attention should be paid to avoid contamination of endoscopic vision.

## Figures and Tables

**Table 1:** Comparison of the general data among the two groups.

	Control group (*n* = 58)	CN injection group (*n* = 50)	*P* value
Sex			
Male	16	9	0.239
Female	42	41	
Age (‾*χ* ± *s*, yrs)	44.0 ± 10.2	46.8 ± 11.9	0.199
Tumor size (cm)	0.63 ± 0.19	0.61 ± 0.24	0.720
Average number of lymph nodes detected per case (number)	4.72 ± 1.89	6.44 ± 2.08	0.000

**Table 2:** Comparison of the parathyroid gland and RLN injury in two groups.

Group	Number of cases	Detection of PGs case (%)	RLN injury case (%)
Control group	58	16 (27.6%)	4 (6.9%)
CN injection group	50	6 (12%)	3 (6%)
*P* value		0.045	1.0

**Table 3:** Comparison of thyroid and parathyroid functions in two groups.

Control group (*n* = 58)		CN injection group (*n* = 50)	*t*	*P* value
FT_3_ (pmol/L)	4.40 ± 0.82	4.59 ± 0.77	−1.26	0.208
FT_4_ (pmol/L)	12.58 ± 3.54	11.51 ± 2.60	1.76	0.08
TSH (mIU/L)	1.98 ± 1.63	2.01 ± 1.24	−0.12	0.9
PTH (ng/L)	32.41 ± 16.46	33.70 ± 14.29	−0.43	0.66
Serum Ca^2+^ (mmol/L)	2.15 ± 0.09	2.17 ± 0.09	−0.82	0.41

FT_3_, free triiodothyronine; FT_4_, free thyroxine; TSH, thyrotropin-stimulating hormone; PTH, parathyroid hormone; Serum Ca^2+^, serum calcium.

## Data Availability

The datasets used or analyzed during the current study are available from the corresponding author upon request.

## References

[1] Davies L., Welch H. G. (2014). Current thyroid cancer trends in the United States. *JAMA Otolaryngology-Head & Neck Surgery*.

[2] Siegel R. L., Miller K. D., Jemal A. (2019). Cancer statistics, 2019. *CA: A Cancer Journal for Clinicians*.

[3] Pellegriti G., Frasca F., Regalbuto C., Squatrito S., Vigneri R. (2013). Worldwide increasing incidence of thyroid cancer: update on epidemiology and risk factors. *Journal of Cancer Epidemiology*.

[4] Udelsman R., Chen H. (1999). The current management of thyroid cancer. *Advances in Surgery*.

[5] Paterson I. C. M., Greenlee R., Jones A. D. (1999). Thyroid cancer in Wales 1985-1996: a cancer registry-based study. *Clinical Oncology*.

[6] Siegel R. L., Miller K. D., Jemal A. (2017). Cancer statistics, 2017. *CA: A Cancer Journal for Clinicians*.

[7] Wittekind C., Compton C. C., Greene F. L., Sobin L. H. (2002). TNM residual tumor classification revisited. *Cancer*.

[8] Hughes D. T., Doherty G. M. (2011). Central neck dissection for papillary thyroid cancer. *Cancer Control*.

[9] Takami H., Ito Y., Okamoto T., Yoshida A. (2011). Therapeutic strategy for differentiated thyroid carcinoma in Japan based on a newly established guideline managed by Japanese society of thyroid surgeons and japanese association of endocrine surgeons. *World Journal of Surgery*.

[10] Yang F., Jin C., Yang D. (2011). Magnetic functionalised carbon nanotubes as drug vehicles for cancer lymph node metastasis treatment. *European Journal of Cancer*.

[11] Hao R. T., Chen J., Zhao L. H. (2012). Sentinel lymph node biopsy using carbon nanoparticles for Chinese patients with papillary thyroid microcarcinoma. *European Journal of Surgical Oncology (EJSO)*.

[12] Sun S. P., Zhang Y., Cui Z. Q. (2014). Clinical application of carbon nanoparticle lymph node tracer in the VI region lymph node dissection of differentiated thyroid cancer. *Genetics and Molecular Research*.

[13] Zhu Y., Chen X., Zhang H. (2016). Carbon nanoparticle-guided central lymph node dissection in clinically node-negative patients with papillary thyroid carcinoma. *Head & Neck*.

[14] Huang K., Luo D., Huang M., Long M., Peng X., Li H. (2013). Protection of parathyroid function using carbon nanoparticles during thyroid surgery. *Otolaryngology-Head and Neck Surgery*.

[15] Zhang Z., Wang Y. (2013). Is carbon nanoparticle useful in thyroid surgery regardless of surgery extent and experience?. *Otolaryngology–Head and Neck Surgery*.

[16] Hüscher C. S., Chiodini S., Napolitano C., Recher A. (1997). Endoscopic right thyroid lobectomy. *Surgical Endoscopy*.

[17] Ikeda Y., Takami H., Sasaki Y., Kan S., Niimi M. (2000). Endoscopic neck surgery by the axillary approach. *Journal of the American College of Surgeons*.

[18] Ohgami M., Ishii S., Arisawa Y. (2000). Scarless endoscopic thyroidectomy: breast approach for better cosmesis. *Surgical Laparoscopy, Endoscopy & Percutaneous Techniques*.

[19] Linos D. (2011). Minimally invasive thyroidectomy: a comprehensive appraisal of existing techniques. *Surgery*.

[20] Yang Y., Gu X., Wang X., Xiang J., Chen Z. (2012). Endoscopic thyroidectomy for differentiated thyroid cancer. *The Scientific World Journal*.

[21] Hughes D. T., Haymart M. R., Miller B. S., Gauger P. G., Doherty G. M. (2011). The most commonly occurring papillary thyroid cancer in the United States is now a microcarcinoma in a patient older than 45 years. *Thyroid*.

[22] Wang Y., Liu K., Xiong J., Zhu J. (2015). Total endoscopic versus conventional open thyroidectomy for papillary thyroid microcarcinoma. *Journal of Craniofacial Surgery*.

[23] Chen C., Huang S., Huang A. (2018). Total endoscopic thyroidectomy versus conventional open thyroidectomy in thyroid cancer: a systematic review and meta-analysis. *Therapeutics and Clinical Risk Management*.

[24] Wang B., Qiu N.-C., Zhang W. (2015). The role of carbon nanoparticles in identifying lymph nodes and preserving parathyroid in total endoscopic surgery of thyroid carcinoma. *Surgical Endoscopy*.

[25] Sywak M., Cornford L., Roach P., Stalberg P., Sidhu S., Delbridge L. (2006). Routine ipsilateral level VI lymphadenectomy reduces postoperative thyroglobulin levels in papillary thyroid cancer. *Surgery*.

[26] Tisell L.-E., Nilsson B., Mölne J. (1996). Improved survival of patients with papillary thyroid cancer after surgical microdissection. *World Journal of Surgery*.

[27] Ji Y. B., Lee K. J., Park Y. S., Hong S. M., Paik S. S., Tae K. (2012). Clinical efficacy of sentinel lymph node biopsy using methylene blue dye in clinically node-negative papillary thyroid carcinoma. *Annals of Surgical Oncology*.

[28] Carcoforo P., Feggi L., Trasforini G. (2007). Use of preoperative lymphoscintigraphy and intraoperative gamma-probe detection for identification of the sentinel lymph node in patients with papillary thyroid carcinoma. *European Journal of Surgical Oncology (EJSO)*.

[29] Raijmakers P. G. H. M., Paul M. A., Lips P. (2008). Sentinel node detection in patients with thyroid carcinoma: a meta-analysis. *World Journal of Surgery*.

[30] Fewins J., Simpson C. B., Miller F. R. (2003). Complications of thyroid and parathyroid surgery. *Otolaryngologic Clinics of North America*.

[31] Youngwirth L., Benavidez J., Sippel R., Chen H. (2010). Parathyroid hormone deficiency after total thyroidectomy: incidence and time. *Journal of Surgical Research*.

[32] Lin D. T., Patel S. G., Shaha A. R., Singh B., Shah J. P. (2002). Incidence of inadvertent parathyroid removal during thyroidectomy. *The Laryngoscope*.

[33] Sorgato N., Pennelli G., Boschin I. M. (2009). Can we avoid inadvertent parathyroidectomy during thyroid surgery?. *In Vivo*.

